# Unraveling the complexities of circadian and sleep interactions with memory formation through invertebrate research

**DOI:** 10.3389/fnsys.2014.00133

**Published:** 2014-08-04

**Authors:** Maximilian Michel, Lisa C. Lyons

**Affiliations:** ^1^Department of Molecular Physiology and Biophysics, Vanderbilt University School of MedicineNashville, TN, USA; ^2^Department of Biological Science, Program in Neuroscience, Florida State UniversityTallahassee, FL, USA

**Keywords:** circadian rhythms, learning and memory, sleep, *Aplysia*, invertebrates

## Abstract

Across phylogeny, the endogenous biological clock has been recognized as providing adaptive advantages to organisms through coordination of physiological and behavioral processes. Recent research has emphasized the role of circadian modulation of memory in generating peaks and troughs in cognitive performance. The circadian clock along with homeostatic processes also regulates sleep, which itself impacts the formation and consolidation of memory. Thus, the circadian clock, sleep and memory form a triad with ongoing dynamic interactions. With technological advances and the development of a global 24/7 society, understanding the mechanisms underlying these connections becomes pivotal for development of therapeutic treatments for memory disorders and to address issues in cognitive performance arising from non-traditional work schedules. Invertebrate models, such as *Drosophila melanogaster* and the mollusks *Aplysia* and *Lymnaea*, have proven invaluable tools for identification of highly conserved molecular processes in memory. Recent research from invertebrate systems has outlined the influence of sleep and the circadian clock upon synaptic plasticity. In this review, we discuss the effects of the circadian clock and sleep on memory formation in invertebrates drawing attention to the potential of *in vivo* and *in vitro* approaches that harness the power of simple invertebrate systems to correlate individual cellular processes with complex behaviors. In conclusion, this review highlights how studies in invertebrates with relatively simple nervous systems can provide mechanistic insights into corresponding behaviors in higher organisms and can be used to outline possible therapeutic options to guide further targeted inquiry.

## Scope of problem

In the past century, the nature of human society has been dramatically altered by technological innovations, communication advances, transportation improvements and urbanization. Non-traditional work schedules and round-the-clock manufacturing shifts have become increasingly common worldwide with the proportion of individuals working non-traditional work schedules rising. Recent research indicates approximately 3.7% of employed adult workers in the United States work a night shift with an additional 23.5% of individuals working non-traditional shifts including evening, rotating or split shifts (Luckhaupt, 2012; Alterman et al., 2013). Irregular work hours or jet lag desynchronize internal circadian oscillators that function to coordinate metabolic, physiological and behavioral processes in anticipation of daily environmental changes and orchestrate the timing of physiological and metabolic processes with behavioral activities.

Although the core circadian oscillator functions independently within individual cells, synchronization between oscillatory neurons and pacemakers is necessary to form functional circadian clocks for tissue and systems level rhythmicity (Albrecht, 2012). Neurons within the suprachiasmatic nucleus (SCN) are traditionally considered to comprise the master circadian clock in mammals. However, glial cells also have functional circadian oscillators and may modulate neuronal regulation of output rhythms (Prolo et al., 2005); for a review see Jackson (2011). Outside of the SCN, independent central circadian oscillators function within the olfactory bulb (Granados-Fuentes et al., 2004a, b, 2006) and rhythmic gene expression can be observed in multiple brain regions including the hippocampus (Holmes et al., 1995; Schaaf et al., 2000; Li et al., 2013). In mammals, as well as in lower vertebrates such as zebrafish (Whitmore et al., 2000) or invertebrates like *Drosophila* (Plautz et al., 1997), self-sustaining peripheral circadian oscillators can be found across multiple cell types and tissues including liver, heart, kidney, adrenal gland, pancreas and even fibroblasts (Balsalobre et al., 2000; Yoo et al., 2004), for reviews see Albrecht (2012) and Brown and Azzi (2013). These peripheral oscillators may be entrained at variable rates or through mechanisms in addition to SCN signaling such as the time of food intake, body temperature, or metabolite and hormonal signaling (reviewed in Dibner et al., 2010; Albrecht, 2012; Mohawk et al., 2012), confounding the necessary resynchronization of oscillators between multiple tissues following jet-lag, irregular work hours or behaviors.

Technological advances have created the phenomenon of social jet lag for many age groups in which individuals significantly shift their wake/sleep cycles on weekends compared to the work week resulting in a bi-weekly activity dependent phase-shifting of the circadian clock (Wittmann et al., 2006; Roenneberg, 2013). Adolescents, with their circadian rhythms developmentally shifted toward late night chronotypes in particular are susceptible to social jet lag, which can result in negative health consequences and cognitive decrements (Collado Mateo et al., 2012; Touitou, 2013; Haraszti et al., 2014). The rising trends in the number of individuals affected by shift work, technological advances and social jet lag have resulted in an increasing proportion of the population that can be considered to have circadian dysfunction.

At the level of the individual, career and societal pressures often result in longer work days with extended temporal demands for high performance leaving less time for rest (Knutson et al., 2010; Roenneberg, 2013). Based on self-reported data collected through national surveys in 2005 and 2010, one-third of adult U.S. workers (approximately 40.6 million individuals) sleep 6 h or less at night (Cdc, C.F.D.C.a.P., 2005, 2007–2010). Although some differing conclusions exist between studies, in general it appears that individuals in the 21st century get significantly less rest compared to individuals 50–80 years ago, with rest time continuing to decrease over the last decade (Knutson et al., 2010; Bin et al., 2012; Luckhaupt, 2012; Roenneberg, 2013). Thus, the problems of sleep restriction, sleep disorders and circadian dysfunction appear pervasive in modern society.

Disturbances of the circadian clock through desynchronization or circadian dysfunction result in increased health problems for individuals with increased risk and incidence of metabolic diseases such as obesity and diabetes, cancer and heart disease as well as many other conditions (Barnard and Nolan, 2008; Preuss et al., 2008; Arble et al., 2010; Bass and Takahashi, 2010; Karatsoreos et al., 2011; Evans and Davidson, 2013; Orozco-Solis and Sassone-Corsi, 2014; Pluquet et al., 2014). Restricted sleep and sleep disorders also adversely impact individual health through increased risk of cardiovascular disease, immune system disorders, emotional and mood disorders, increased susceptibility to metabolic disorders, decreased cognitive performance and reduced quality of life and well-being (Breslau et al., 1996; Suka et al., 2003; Burgos et al., 2006; Neckelmann et al., 2007; Benca and Peterson, 2008; Goel et al., 2009; Vgontzas et al., 2009; Leproult and Van Cauter, 2010; Hsieh et al., 2011; Grandner et al., 2012). The increased incidence of automobile accidents, industrial accidents and occupational errors associated with sleep deprivation and sleep disorders raises the issue from the level of the individual to a societal crisis (Horne and Reyner, 1995; Pack et al., 1995; Lyznicki et al., 1998; Landrigan et al., 2004; Barger et al., 2005). Furthermore, decreased worker productivity leads to economic costs for businesses and industries with increased health problems exacerbating the loss in productivity by increasing health care costs to both employees and employers.

Independently of sleep deprivation or circadian dysfunction, the circadian clock itself modulates memory in humans and results in optimal times for memory and performance as well as trough phases in which significantly decreased performance in cognitive tasks occurs (Wright et al., 2002, 2006, 2012; Goel et al., 2013). This compounds the problems associated with shift work as circadian misalignment exists between the optimal times for cognitive performance and the phase in which some work is being performed. Moreover, when the effects of circadian phase such as work during a rest phase are compounded with extended time awake, the negative impacts on human cognitive performance are magnified (Dijk et al., 1992; Silva et al., 2010; Matthews et al., 2012b). Thus, the sleep restriction and fatigue faced by night shift workers, individuals in the transportation industry or health professionals, increase the problems associated with trying to optimize performance during sub-optimal circadian phases for cognitive performance. Additionally, the circadian clock can also modulate the impact of sleep deprivation on cognitive performance, further exacerbating the problem (Lo et al., 2012; Matthews et al., 2012a).

Despite the tremendous impact of sleep disorders and circadian dysfunction on individual health and society, the mechanisms and tri-partite interactions between sleep, the circadian clock and memory remain ill-defined at the synaptic or neuronal level. There is a continuing need for basic research investigating the underlying neural and molecular architecture involved in sleep, memory and circadian interactions in order to develop future treatments for disorders, improve cognitive performance, or design strategies to cope with the problems of shift work and social jet lag. Invertebrate research has proven to be fundamental in illuminating the basic principles and mechanisms underlying sleep, circadian rhythms and neuronal plasticity individually. In this review we will highlight recent advances that provide the framework and first steps to elucidate the interplay of these three research areas and discuss potential future directions.

## Invertebrates in neurobiology research

Given the considerable neuroanatomical differences between invertebrate models for neuroscience research (arthropods and mollusks) and higher organisms, differences in the levels of behavioral complexity, and the recent technological advances for targeted genetic studies in mammals, the reader may wonder whether research using invertebrate model systems will continue to advance progress in neuroscience research. Although vertebrate and invertebrate lineages diverged more than 900 million years ago (Peterson et al., 2004), the molecular and cellular mechanisms underlying neuronal behavior and synaptic plasticity are surprisingly well conserved across phylogeny. Understanding complex behaviors and the underlying cellular and molecular mechanism in higher organisms can be significantly facilitated through the study of these processes in comparatively less complex organisms.

Model systems such as *Drosophila melanogaster* and *Caenorhabditis elegans* have harnessed the power of neurogenetics to dissect mechanisms underlying behavior. For example, research in *Drosophila* was key in identifying mechanisms underlying the core circadian oscillator (reviewed in Allada and Chung, 2010; Hardin, 2011; Ozkaya and Rosato, 2012) as well as the identification of signaling pathways underlying associative memory formation (McGuire et al., 2005; Davis, 2011) to name just two examples. *C. elegans* has furthermore emerged as a genetic model for studying memory and aging (reviewed in Murakami, 2007; Stein and Murphy, 2012; Chen et al., 2013; Sasakura and Mori, 2013). The tractability of these invertebrate model systems has been enhanced by powerful neurogenetic techniques that include forward genetic screens, reverse genetic techniques with genome-wide RNAi lines available, and optogenetics to assess individual neuronal changes using voltage or calcium sensors (reviewed in Sattelle and Buckingham, 2006). Recent research in alcohol neurobiology and drug addiction using *Drosophila* has demonstrated how insights gained from invertebrates can be leveraged into rapid advances in mammalian systems (Corl et al., 2009; Kaun et al., 2012; Kapfhamer et al., 2013). Likewise, in sleep studies, research using invertebrate models has advanced research in higher organisms as was elegantly shown by Paul Shaw and colleagues. In these studies, candidate genes for putative biomarkers of sleep loss were identified in human and rodent models and tested in *Drosophila*, subsequently facilitating further studies of additional biomarkers in mammals (Thimgan et al., 2013). The tools available for invertebrate research provide cost-effective, experimentally tractable systems for the rapid identification of novel pathways and cellular interactions associated with defined behaviors that can subsequently be investigated in more complex model systems.

The large size, determinate neuron position and the relatively small number of neurons in Molluscan species such as the marine mollusk *Aplysia californica* have proven invaluable for characterizing changes in cellular signaling pathways and synaptic plasticity associated with memory formation (Kandel, 2001; Bailey et al., 2008; Kandel et al., 2014). Likewise, studies in the freshwater pond snail *Lymnaea stagnalis* have led to important insights into the mechanisms of memory formation, particularly for the modulation of memory (Kemenes et al., 2006; Marra et al., 2013; Lukowiak et al., 2014). Moreover, these invertebrates have been pivotal in the initial recognition of non-synaptic forms of neuronal plasticity and their possible role in the neuronal representation of memory (Mozzachiodi and Byrne, 2010; Nikitin et al., 2013). Lastly, studies of neuronal injury and plasticity using molluscan models have expanded our understanding of chronic pain and other neurological disorders in humans (reviewed in Weragoda and Walters, 2007; Walters and Moroz, 2009; Crook et al., 2013).

Thus, research in invertebrates provides the ability to study system level interactions with broad impacts throughout the organism on physiological, behavioral and metabolic processes, such as the circadian clock, sleep, drug and alcohol use and neurological diseases. Although differing biological and evolutionary constraints may give rise to vital differences between invertebrate and vertebrate systems, an understanding of how evolution solved essential and complex conserved phenomena such as the interplay between memory and sleep in “simple” organisms will provide crucial insight into the molecular and cellular building blocks underlying these phenotypes in man.

## Sleep in invertebrates

To unravel the interactions between the circadian clock, sleep and memory formation, it is necessary to have a model system in which all three processes interact. The repeated appearance of sleep across phylogenies suggests that sleep is an evolutionary necessity and its functions are conserved, if not its origins. In mammals and birds, sleep consists of two main stages, slow wave sleep and rapid eye movement sleep (REM), characterized by changes in neuronal activity as measured by electroencephalograms (reviewed in Rattenborg, 2006; Madan and Jha, 2012). Despite the similarities in the two stage sleep state of these groups, the emergence of REM and NREM sleep appears to be through convergent evolution (Rattenborg, 2007; Rattenborg et al., 2012). In aquatic mammals, reptiles, amphibians and other vertebrates, slow wave sleep has been consistently detected although REM sleep is not always present (Hobson et al., 1968; Mukhametov et al., 1977; González et al., 1999) reviewed in Williams et al. (1973) and Madan and Jha (2012). Monotremes, such as the echidna and platypus, demonstrate a single sleep state that shows characteristics of both slow wave and REM sleep (Siegel et al., 1996, 1998, 1999; Nicol et al., 2000). The uni-hemispheric presentation of sleep such as observed in dolphins (Mukhametov et al., 1977; Mukhametov, 1987; Sekiguchi and Kohshima, 2003) or the appearance of local sleep in sub-regions of the brain may explain the absence or minimization of REM sleep in aquatic mammals and other non-mammalian vertebrates (reviewed in Madan and Jha, 2012; Rattenborg et al., 2012). Local sleep with concurrent changes limited to specific neuronal groups complicates the investigation of the mechanisms underlying the interactions of sleep, the circadian clock and synaptic plasticity or memory formation in higher organisms. As a first step, it is necessary to study all components within the same circuit or neuronal network making the lower complexity of invertebrates attractive for research. However, this raises the question “do invertebrates sleep in an analogous manner to higher organisms?”

Invertebrate sleep is defined by means of behavioral characteristics including rhythmic activity and rest behaviors, characteristic rest body posture, preferred resting location, decreased responsiveness to sensory stimuli during rest (increased arousal thresholds) and rebound following rest deprivation (homeostasis) (Tobler, 1983; Hendricks et al., 2000; Zimmerman et al., 2008). As the regulation of sleep occurs dually through homeostatic processes and the circadian clock (Borbély and Achermann, 1999), interaction of the rest state with the circadian clock or expression of clock genes may also be used to further define sleep. Using some or all of these criteria, sleep or sleep-like states have been identified in dozens of invertebrates across phyla. Table 1 highlights the activity phase and the type of sleep regulation observed for select invertebrate models that have also been used as models for studies of learning and memory or circadian research.

**Table 1:** **Examples of invertebrate species from the Phyla Mollusca and Arthropoda in which sleep has been studied**.

	**Organism**	**Activity Phase**	**Sleep characteristic**	**Reference**
**Phylum Mollusca**	*Aplysia*	Diurnal	Homeostatic and Circadian Regulation	(Strumwasser, 1973; Vorster et al., in press)
	*Lymnaea stagnalis* (freshwater pond snail)	Greater Activity During Early Day	Sporadic Bouts, Regulation Unknown	(Wagatsuma et al., 2004; Aono et al., 2008; Stephenson and Lewis, 2011)
	*Octopus*	Nocturnal with pronounced dawn/dusk activity; may vary	Homeostatic and Circadian Regulation	(Brown et al., 2006; Meisel et al., 2006; Hochner, 2010)
	*Sepia* (Cuttlefish)	Diurnal, may vary	Homeostatic Regulation, Multiple Sleep-like States	(Duntley et al., 2002; Hanlon et al., 2007; Frank et al., 2012)
**Phylum Arthropoda**	*Drosophila melanogaster* (fruitfly)	Diurnal; Crepuscular	More sleep at night; Homeostatic and Circadian Regulation	(Hendricks et al., 2000; Shaw et al., 2000; van Alphen et al., 2013) reviewed in Bushey and Cirelli (2011) and Potdar and Sheeba (2013)
	*Apis mellifera* (honeybee)	Diurnal	Varies with worker caste and age; Multiple sleep stages; Homeostatic and Circadian Regulation	(Kaiser and Steiner-Kaiser, 1983; Kaiser, 1988; Sauer et al., 2003, 2004; Eban-Rothschild and Bloch, 2008, 2012; Klein et al., 2008)
	*Leucophaea maderae; Blaberus giganteus* (cockroach)	Nocturnal	Homeostatic and Circadian Regulation	(Tobler, 1983; Tobler and Neuner-Jehle, 1992; Decker et al., 2007; Garren et al., 2013)
	*Procambrus clarkii* (crayfish)	Diurnal	Slow wave brain activity during sleep, Homeostatic regulation	(Ramón et al., 2004; Mendoza-Angeles et al., 2010; Ramon et al., 2012)

Recent advances using these relatively simple invertebrate systems have identified molecular and circuit mechanisms underlying sleep. For example, a sleep-like state has been reported for larval and adult stages of the nematode *C. elegans* in which quiescence is characterized by reduced sensory responsiveness, a characteristic rest body posture, and is timed through the expression of an ortholog of the canonical clock gene *period* (Jeon et al., 1999; You et al., 2008; Zimmerman et al., 2008; Iwanir et al., 2013; Nelson and Raizen, 2013). The simple wiring of the *C. elegans* nervous system allowed the identification of neuronal changes in sensory neurons and interneurons underlying the decreased arousal and quick reversibility of quiescence (Schwarz et al., 2011; Cho and Sternberg, 2014). At the molecular level, regulation of this sleep-like behavior has been associated with changes in the expression of highly conserved transcription factors (Driver et al., 2013; Turek et al., 2013) and neuropeptide signaling (Nelson and Raizen, 2013). The rapidity of research advances in the identification of the molecular and cellular mechanisms underlying sleep in *C. elegans* highlights the usefulness and potential for simple invertebrates in sleep research.

In insects, detailed studies of sleep have been performed in many species including cockroaches, bees, mosquitoes and *Drosophila* (Tobler, 1983; Tobler and Neuner-Jehle, 1992; Hendricks et al., 2000; Shaw et al., 2000; Eban-Rothschild and Bloch, 2008; Klein et al., 2008; Bushey et al., 2011). Over the past 15 years, our understanding of the functions of sleep and its functional necessity has rapidly progressed through research in *Drosophila* (reviewed in Cirelli and Tononi, 2008; Piscopo, 2009; Potdar and Sheeba, 2013) by providing evidence for the synaptic homeostasis hypothesis of sleep proposed by Tononi and Cirelli (2003). The synaptic homeostasis hypothesis is similar to synaptic scaling or homeostatic plasticity proposed for learning and memory (for a recent review see Schacher and Hu, 2014) and suggests that periods of activity with concomitant increases in synaptic strength are followed by synaptic downscaling during periods of sleep including decreases in synapse number (synaptic pruning) and weakening of synaptic connections (reviewed in Tononi and Cirelli, 2006, 2012). Structural evidence for synaptic pruning during sleep was recently provided in *Drosophila* (Bushey et al., 2011) along with the identification of sleep/wake changes in synaptic markers (Gilestro et al., 2009) consistent with the synaptic homeostasis hypothesis. *Drosophila* as a model has also been invaluable in advancing our understanding of the role of experience dependent plasticity in the need for sleep (Donlea et al., 2009) and the role of sleep in memory consolidation (Donlea et al., 2011). Furthermore, sleep in *Drosophila* also occurs in different stages with a deeper intensity sleep stage evident through recordings of brain activity (van Alphen et al., 2013). The rapid generation time of *Drosophila* combined with powerful neurogenetic approaches and forward genetic screens have allowed researchers to identify numerous genes and pathways regulating arousal and sleep (Koh et al., 2008; Chen et al., 2014; Park et al., 2014; Shi et al., 2014; Wu et al., 2014) as well as the timing of sleep onset (Liu et al., 2014). These factors have propelled *Drosophila* to the forefront of sleep research as a future model for screening and thereby understanding the genetic basis for sleep disorders in humans (Freeman et al., 2013). Despite these incredible and rapid advances in sleep research and the relatively small size of the *Drosophila* brain compared to mammalian brains, the complexity of the *Drosophila* nervous system combined with small neuronal size still poses some difficulties in decoding the tripartite interactions of sleep, the circadian clock and memory formation within the same neuronal circuit.

In the morphologically diverse phylum Mollusca, conservation of sleep has been shown with sleep-like states characterized in Cephalopods and Gastropods. Cephalopods are often referred to as advanced invertebrates (Zullo and Hochner, 2011), as they have a highly developed centralized nervous systems and complex behaviors including higher order learning. *Octopus vulgaris* and the cuttlefish *Sepia* demonstrate sleep similar to vertebrate organisms with corresponding changes in behavior and differential brain activity (Duntley et al., 2002; Brown et al., 2006; Frank et al., 2012). However, increasing difficulties of working with cephalopods in laboratory settings and concerns for animal welfare (Fiorito et al., 2014) combined with the complexity of the cephalopod central nervous system make further studies of circadian and sleep interaction with memory or synaptic plasticity more difficult.

In comparison to cephalopods, gastropods are very different in morphological organization and development of the nervous system with a relatively simple central nervous systems and neurons clustered in distributed ganglia. Gastropods, including the terrestrial snail *Helix*, the pond snail *Lymnaea*, and the sea slugs *Hermissenda* and *Aplysia* have long been favorite models for neuroscience research and studies of synaptic plasticity. Anecdotal evidence and isolated literature references in early circadian and behavioral studies suggested resting or quiescent sleep states in *Aplysia* (Strumwasser, 1971, 1973; Preston and Lee, 1973), although systematic analysis of *Aplysia* sleep has only occurred recently. *Aplysia* sleep appears monophasic along circadian time with rest occurring only during the night. *Aplysia* further exhibit decreased sensory responsiveness during rest and sleep rebound following manual rest deprivation (Vorster et al., in press). Although the nervous system of the related gastropod *Lymnaea* *stagnalis* appears similar to *Aplysia*, surprisingly, rest-like states appear to be very different, with rest occurring infrequently and sporadically totaling less than 10% of the time (Stephenson and Lewis, 2011). Moreover, *Lymnaea* sleep does not appear to be regulated by the circadian clock, and rebound rest following deprivation has not been shown (Stephenson and Lewis, 2011; Stephenson, 2011). From these studies one could conclude either that* Lymnaea* require little sleep based upon their ecological niche or that sleep may be regulated via other mechanisms. Alternately, one may hypothesize that behavioral measures of sleep may not always be sufficient to characterize sleep and further investigations in simple invertebrates should be conducted to determine if sleep occurs at the level of the individual neuron. Although detailed characterization of the role of sleep in memory formation and synaptic plasticity has not yet been done in *Lymnaea* or *Aplysia*, the demonstration of sleep in these organisms highlights the potential for future research investigating the function and role of sleep in neuronal plasticity and memory formation using these classic neuroscience models.

## The circadian clock and memory

Energetically, neuronal and synaptic activities are expensive with the brain consuming about 20% of the total energy budget (reviewed in Harris et al., 2012). Behavioral evidence for the higher costs of neuronal activity and synaptic plasticity can be found in *Drosophila* (reviewed in Burns et al., 2011). Long-term memory formation is limited when starvation conditions exist (Plaçais and Preat, 2013) and memory formation decreases the resistance of flies to extreme stress (Mery and Kawecki, 2005). An evolutionary tradeoff exists in *Drosophila* between longevity and memory formation with longer-lived strains showing lower levels of memory (Burger et al., 2008; Lagasse et al., 2012). Likewise, selection of strains of flies with increased long-term memory results in decreases in longevity presumably due to the high metabolic costs associated with the improvements in memory and performance (Burger et al., 2008; Lagasse et al., 2012). One of the underlying tenets of the synaptic homeostasis hypothesis for sleep is that stronger synapses and strengthening of synapses during waking lead to higher energy consumption, that can be compensated for with synaptic downscaling during sleep which permits decreases in energy consumption allowing energy balance and restoration of cellular homeostasis (reviewed in Tononi and Cirelli, 2014). Thus while sleep functions as one mechanism for restoring energy balance, the energy demands associated with memory formation still exert a strong selective pressure for limiting memory formation as can be seen with the longevity studies in *Drosophila*.

One possible mechanism to restrict memory formation in concert with the animal’s activity and physiology is through circadian modulation of memory. The far-reaching impact of the circadian clock on physiology can be observed through the adverse impacts associated with circadian dysfunction or desynchronization. Circadian dysfunction increases the risk of disease incidence for heart disease, metabolic diseases such as diabetes, multiple forms of cancer, mood disorders (reviewed in Barnard and Nolan, 2008; Preuss et al., 2008; Arble et al., 2010; Bass and Takahashi, 2010; Karatsoreos et al., 2011; Evans and Davidson, 2013; Orozco-Solis and Sassone-Corsi, 2014; Pluquet et al., 2014) as well as decrements in cognitive performance (Cho et al., 2000; Gibson et al., 2010; Loh et al., 2010). The broad scope of circadian modulation of metabolism and physiology suggests that there may be multiple levels through which the circadian clock could impact memory formation including sensory gating, enzymatic activity, intracellular signaling cascades, macromolecular synthesis or epigenetic regulation.

Interestingly, the mechanisms through which the circadian clock impacts memory appears to depend upon the type of learning, e.g., non-associative, classical or operant, and the type of memory formed, e.g., short, intermediate or long-term memory. Furthermore, the circadian clock may target memory through its induction, molecular consolidation, and/or recall. Focusing on invertebrate research, circadian modulation of memory has been observed in both insects and mollusks (see Table 2 for examples). In cockroaches, short and long-term memories induced through classical olfactory conditioning are highly dependent upon time of training (Decker et al., 2007). Interestingly, during operant conditioning the circadian clock modulates recall, rather than memory formation, demonstrating how the circadian clock may regulate different steps in memory (Garren et al., 2013). In *Drosophila*, circadian modulation of olfactory conditioning has been shown directly for short-term and long-term memory (Lyons and Roman, 2008; Fropf et al., 2014) as well indirectly via circadian modulation of the vulnerability of anesthesia resistant memory to sleep deprivation (Le Glou et al., 2012). A recent study furthermore starts to unravel the molecular mechanisms by implicating circadian changes of a constitutive transcription factor well known for its role in long-term memory (Fropf et al., 2014). Core circadian clock genes also affect long-term memory formation as shown for courtship conditioning (Sakai et al., 2004). In honeybees, learning and memory are also modulated by the circadian timing of training (Lehmann et al., 2011). These studies in insects clearly demonstrate that circadian regulation of memory can occur at multiple stages including the formation and recall of memory.

**Table 2:** **Examples of species frequently used for learning and memory studies in which sleep or circadian modulation of memory has also been examined**.

	**Organism**	**Learning Paradigm**	**Type of Modulation**	**Reference**
**Phylum Mollusca**	*Aplysia*	Sensitization	Circadian modulation of intermediate and long-term memory formation, peak memory during the day	(Fernandez et al., 2003; Lyons et al., 2006a,b, 2008)
		Operant conditioning—feeding behavior	Circadian modulation of intermediate and long-term memory formation, peak memory during the day for diurnal *A. californica*. Peak long-term memory at night for nocturnal species	(Lyons et al., 2005, 2006b; Michel et al., 2013)
		Habituation, Classical Conditioning	Not Examined	Reviewed in Kandel (2001)
	*Lymnaea stagnalis* (pond snail)	Conditioned taste aversion	Diurnal modulation; peak learning in early morning	(Wagatsuma et al., 2004; Stephenson and Lewis, 2011)
	*Octopus*	Avoidance learning, Touch Discrimination, long-term potentiation	Not Examined	(Wells, 1965; Wells and Young, 1965; Hochner et al., 2003; Shomrat et al., 2008; Hochner, 2010)
	*Sepia* (Cuttlefish)	Spatial memory, associative memory	Not Examined	(Duntley et al., 2002; Alves et al., 2007; Frank et al., 2012; Cartron et al., 2013)
**Phylum Arthropoda**	*Drosophila melanogaster* (fruitfly)	Aversive phototactic suppression	Sleep deprivation affects short and long-term memory	(Seugnet et al., 2009, 2011)
		Courtship Conditioning	Sleep deprivation impacts long-term memory; increased sleep enhances long-term memory	(Ganguly-Fitzgerald et al., 2006; Donlea et al., 2011)
		Olfactory memory	Circadian and diurnal modulation of short and long-term memory	(Lyons and Roman, 2008; Fropf et al., 2014)
			Sleep deprivation affects short-term memory and long-term memory	(Li et al., 2009; Le Glou et al., 2012)
		Place preference	Decreased sleep impairs memory	(Bushey et al., 2007)
	*Apis mellifera* (honeybee)	Olfactory memory	Diurnal and circadian modulation of memory; Sleep deprivation affects extinction learning	(Hussaini et al., 2009; Lehmann et al., 2011)
		Spatial memory	Sleep deprivation affects memory consolidation	(Moore and Doherty, 2009; Moore et al., 2011; Beyaert et al., 2012)
	*Leucophaea maderae* (cockroach)	Olfactory memory	Circadian regulation of short and long-term memory; stage of modulation varies between classical and operant paradigms	(Tobler, 1983; Tobler and Neuner-Jehle, 1992; Decker et al., 2007; Garren et al., 2013)
	*Manduca sexta* (hawkmoth)	Olfactory memory	Diurnal regulation of short and intermediate-term memory	(Gage et al., 2013; Gage and Nighorn, 2014)
	*Chasmagnathus* (crab)	Habituation	Non-24 h intervals between training and testing impair long-term memory	(Pereyra et al., 1996)

In addition to the circadian clock itself modulating memory or recall, core circadian genes also function in synaptic plasticity outside of their roles in the circadian oscillator. Independent of its canonical role in the *Drosophila* central pacemaker, the blue-light sensor CRYPTOCHROME (CRY) has the ability to mediate neuronal firing rates in response to light through potassium channel conductance (Fogle et al., 2011). Furthermore, the core clock protein PERIOD (PER) appears necessary for robust long-term memory as *per* mutant flies exhibit deficits in conditioned courtship memory (Sakai et al., 2004). Similar complexity in circadian regulation of memory at multiple levels has also been observed in vertebrates (reviewed in Gerstner and Yin, 2010; Jilg et al., 2010; Bechstein et al., 2014).

Circadian modulation of memory dependent upon the time of training has also been shown for intermediate and long-term memory in mollusks where the most extensive studies have been conducted in *Aplysia* (reviewed in Lyons, 2011). Using a non-associative form of learning, sensitization of the tail-siphon withdrawal reflex, it was found that both protein synthesis dependent intermediate-term memory and long-term memory were regulated by the circadian clock dependent upon time of training (Fernandez et al., 2003; Lyons et al., 2008). Although long-term memory in this case appears to be regulated only during its induction and formation, multiple processes are regulated by the circadian clock following training including neurotransmitter release, the induction of MAPK signaling and immediate early gene expression (Lyons et al., 2006a). The circadian clock also modulates intermediate and long-term memory for an associative operant learning paradigm (Lyons et al., 2005; Michel et al., 2013). In contrast to the circadian regulation of short-term memory observed in insects, *Aplysia* short-term memory for either associative or non-associative paradigms does not appear to be regulated by the circadian clock (Fernandez et al., 2003; Lyons et al., 2005), perhaps due to the specific ethological relevance of the learning paradigms used in these studies, a defensive reflex and feeding behavior. In *Lymnaea*, time of day also affects the acquisition of memory for conditioned taste aversion (Wagatsuma et al., 2004). The appearance of strong circadian modulation of memory across species and learning paradigms demonstrates that this modulation provides a clear evolutionary advantage. Recently, studies in rodents have shown that the reverse modulation also occurs, i.e., that memory formation can impact the circadian timekeeping of the organism. Using a hippocampal dependent learning paradigm and sustained activity tasks, researchers demonstrated that training inducing memory formation conducted during the animal’s inactive period resulted in a phase shift in locomotor activity (Gritton et al., 2012). Although the potential bidirectional interaction of memory formation and synaptic activity on the phase of the circadian clock have not been investigated using invertebrate models, understanding the impact of memory formation anti-phase to activity cycles will be important for managing the issues of memory and performance associated with shift work. Thus, it will be of considerable interest to identify the molecular mechanisms of this interaction as well as the parameters and circumstances under which these bi-directional influences occur. Invertebrate models provide excellent systems for investigating the forward modulation of memory by the circadian clock and identifying the types and mechanisms through which memory or cognitive activity impact the circadian oscillator or other circadian rhythms.

## Future directions

Identifying the individual neurons and neural networks causal to complex behaviors and studying the interactions between multiple processes such as memory, sleep and the clock is one of the greatest challenges in modern neuroscience. Using invertebrate model systems, it becomes feasible to investigate the interactions of sleep, the circadian clock and synaptic plasticity at the level of individual neurons or neuronal circuits permitting the possibility of addressing the following topics: (1) determine how individual neurons or neuronal circuits involved in memory formation are stabilized through time, reactivated, undergo periods of malleability and degradation/pruning; (2) determine how the effects of sleep as a network property or at the level of individual neurons affects neuronal plasticity; (3) determine how the core circadian clock regulates sleep in individual neurons and circuits; (4) determine how sleep and circadian time modulate neuronal and synaptic activity for specific tasks or memories; (5) determine how effects analogous to modern life such as non-traditional sleep/wake cycles or shifted circadian rhythms affect this interplay transiently and chronically; and ultimately; (6) identify mechanisms through which these interactions can be modified by drugs or optimized behavioral patterns.

In support of this, recent studies in invertebrates have identified specific neurons that regulate sleep (Donlea et al., 2014) as well as a circadian transcript that modulates sleep onset in *Drosophila* (Liu et al., 2014). Sleep has also been shown to regulate net synaptic homeostasis, thereby “resetting” the nervous system to allow future plasticity (Tononi and Cirelli, 2014). From this and the studies mentioned in the previous sections it follows that the molecular and cellular substrate for the interplay between memory, sleep and the clock is slowly crystallizing from invertebrate studies, and this will significantly inform follow up research in mammalian and simian species.

One of the greatest benefits of the molluscan model system is the spectrum of techniques permitting an integration of top-down (*in-vivo*) and bottom-up (*in vitro*) approaches in the experimental design and the exquisite detail possible with investigations at the level of individual neurons or neuronal circuits. In gastropod mollusks, the precisely wired nervous system is comprised of large neurons with determinate location and in many cases functions of individual neurons identified (see for example Benjamin et al., 2000; Moroz, 2000; Kandel, 2001; Kandel et al., 2014). In models such as *Lymnaea* and *Aplysia*, the advantages of multilevel approaches combining *in vivo* behavioral studies with *in vitro* cellular studies to correlate complex behaviors with individual neuronal behavior have been well-documented.

For example in *Aplysia*, long-term facilitation provides a cellular correlate for behavioral sensitization that can be investigated in reduced preparations and cultured neurons (see for example Kandel, 2001; Hawkins et al., 2006). In a semi-intact reduced preparation in which the siphon and tail are exposed with the nerves connecting the ganglia left intact, intermediate and long-term sensitization can be studied for either the tail-siphon withdrawal reflex or for a tail-tail withdrawal reflex (Philips et al., 2006, 2011). The existence of extra-ocular photoreceptors and extra-ocular circadian oscillators involved in circadian modulation of memory in *Aplysia* (Lyons et al., 2006b) widens the potential for studying system level interactions between the circadian clock and memory formation using semi-intact reduced or whole ganglia preparations. Furthermore, sleep has been characterized in *Aplysia* that demonstrates changes in sensory arousal accompanying sleep and homeostatic rebound rest following sleep deprivation (Vorster et al., in press). Individual neuronal changes correlated with behavioral changes can be identified using well-developed whole mount immunohistochemical and *in situ* techniques as shown in *Aplysia* (Sweedler et al., 2002; Jezzini et al., 2005). The neurons known to underlie behavioral effects seen *in vivo* as well as in the reduced preparation can further be co-cultured in a dish (Cai et al., 2012), or a compartmentalized dish to separately investigate synaptic vs. somatic changes within the neurons (Ye et al., 2012). Moreover, the large size of *Aplysia* neurons and determinate neuronal position also allows for genomic and metabolomic analysis at the scale of the individual neuron (Moroz and Kohn, 2013; Nemes et al., 2013), or even the synaptic transcriptome (Puthanveettil et al., 2013).

Similar anatomical advantages for studies of individual neurons or neuronal circuits exist in *Lymnaea* in which multi-electrode arrays have been used in order to record from up to 60 electrodes simultaneously (Harris et al., 2010). Additionally, the large neuronal size permits investigation of plasticity at subcellular levels using behavioral, electrophysiological and optical recording techniques. For example, recent research in *Lymnaea* demonstrated that learning-induced non-synaptic plasticity contributed to specific, compartmentalized presynaptic changes through calcium signaling (Nikitin et al., 2013). The advantages of large neuronal size and the potential for individual neuronal manipulations through pharmacological or electrophysiological methods combined with the diversity of recording techniques ensures that molluscan neuronal circuits will remain at the forefront of our progress in studies of synaptic plasticity and neuronal behavior.

Given the availability of abundant data underlying neuronal network function in molluscs, mathematical modeling can be leveraged to drive further experimental inquiry (Vavoulis et al., 2007; Liu et al., 2013). Importantly, the incorporation of predictive neuronal networks to direct experimental investigations facilitates the identification of key signaling molecules in behavior and methods to ameliorate conditions caused by mutations in these pathways. In a set of elegant studies, the Byrne lab has modeled the molecular underpinnings of *Aplysia* memory processes to develop a training paradigm predicted to lead to a stronger induction of the molecular cascades necessary for memory. Testing these predictions behaviorally, they found that the modeled training protocols resulted in stronger and longer lasting memory (Zhang et al., 2011). In subsequent studies, the power of computer simulations was leveraged to predict an ameliorated training paradigm to overcome the knockdown of a protein commonly mutated in Rubinstein-Taybi syndrome, strongly suggesting that modeling using the molecular and circuit level insight gained from invertebrates can be used to predict optimal learning strategies potentially in lieu of or in conjunction with drug therapy (Liu et al., 2013).

The multi-level combinatorial approaches outlined above set the stage to yield unprecedented insight into the mechanisms through which sleep or the circadian clock affects neuronal plasticity. The high degree of phylogenetic conservation as previously discussed permits the rapid translation of research in invertebrate systems to mammalian models (see also Abrams, 2012). It stands to reason that even with evolutionary differences resulting in non-conserved transcripts between invertebrates and mammals, the role that these molecules play will have an analogous function in mammals since the circadian clock, sleep and memory are evolutionarily well-conserved processes. Taken together, research in relatively simple mollusks such as *Aplysia* presents the scientific community with a powerful toolset to identify the cellular and molecular pathways from the whole animal to mathematical models, laying the foundation to identify the basic principles and mechanisms of memory modulation. Unraveling the intricate relationships between sleep, the circadian clock and learning and memory is essential to identify key molecules or interacting nodes for the development of future therapies to improve cognitive performance and decrements associated with shift work, sleep disorders, aging or neurological disorders.

## Conflict of interest statement

The authors declare that the research was conducted in the absence of any commercial or financial relationships that could be construed as a potential conflict of interest.
